# A 3D Approach Using a Control Algorithm to Minimize the Effects on the Healthy Tissue in the Hyperthermia for Cancer Treatment

**DOI:** 10.3390/e25040684

**Published:** 2023-04-19

**Authors:** Gustavo Resende Fatigate, Marcelo Lobosco, Ruy Freitas Reis

**Affiliations:** 1 Pós-Graduação em Modelagem Computacional, Universidade Federal de Juiz de Fora, Rua José Lourenço Kelmer, s/n-São Pedro, Juiz de Fora 36036-900, MG, Brazil; 2 Departamento de Ciência da Computação, Universidade Federal de Juiz de Fora, Rua José Lourenço Kelmer, s/n-São Pedro, Juiz de Fora 36036-900, MG, Brazil

**Keywords:** hyperthermia, cancer, bioheat, CUDA, optimization, differential evolution

## Abstract

According to the World Health Organization, cancer is a worldwide health problem. Its high mortality rate motivates scientists to study new treatments. One of these new treatments is hyperthermia using magnetic nanoparticles. This treatment consists in submitting the target region with a low-frequency magnetic field to increase its temperature over 43 °C, as the threshold for tissue damage and leading the cells to necrosis. This paper uses an in silico three-dimensional Pennes’ model described by a set of partial differential equations (PDEs) to estimate the percentage of tissue damage due to hyperthermia. Differential evolution, an optimization method, suggests the best locations to inject the nanoparticles to maximize tumor cell death and minimize damage to healthy tissue. Three different scenarios were performed to evaluate the suggestions obtained by the optimization method. The results indicate the positive impact of the proposed technique: a reduction in the percentage of healthy tissue damage and the complete damage of the tumors were observed. In the best scenario, the optimization method was responsible for decreasing the healthy tissue damage by 59% when the nanoparticles injection sites were located in the non-intuitive points indicated by the optimization method. The numerical solution of the PDEs is computationally expensive. This work also describes the implemented parallel strategy based on CUDA to reduce the computational costs involved in the PDEs resolution. Compared to the sequential version executed on the CPU, the proposed parallel implementation was able to speed the execution time up to 84.4 times.

## 1. Introduction

Cancer is one of the leading causes of death worldwide: this disease was responsible for 10 million deaths in 2020 [1]. Most of those deaths occur among people older than 50 years. The most common types of cancer are breast cancer, lung cancer, and colon and rectum cancer [1,2].

Due to the high mortality rates associated with cancer, new strategies are being developed to fight this disease. One such strategy is hyperthermia, an adjuvant technique used with existing treatments such as radiotherapy and chemotherapy [3]. Magnetic nanoparticles can be delivered to the tumor site via intravenous or direct injection into the tissue. When exposed to a low-frequency magnetic field, these nanoparticles produce heat via Brownian and Neelian relaxation [4]. Hyperthermia, therefore, uses magnetic nanoparticles to heat the tumor tissue to over $43{\;}^{\circ }$C. The efficacy of this approach depends on its ability to increase the temperature above $43{\;}^{{}^{\circ}}$C throughout the tumor while minimizing thermal harm to the surrounding healthy tissue.

This study proposed a strategy for quickly identifying the optimal locations for nanoparticle injection. Specifically, we sought to determine the injection points that will increase the temperature in the tumor tissue to $43{\;}^{{}^{\circ}}$C or higher while minimizing the temperature in surrounding healthy tissue. Throughout this study, we used a target temperature of $T\geq 43{\;}^{{}^{\circ}}$C as the threshold for tissue damage and induction of cell necrosis [5,6,7].

The scientific literature presents several models that have been developed and applied to describe the dynamics of heat in living tissues [8,9,10,11,12]. In this study, we evaluated the heat distribution of the hyperthermia process using Pennes’ model. The low computational cost and reasonable precision of this model justified its choice [5,13,14,15,16,17,18]. The original Pennes equation can be modified to model hyperthermia processes, including the role of magnetic nanoparticles in the bioheat transfer equation [19,20,21,22,23]. We assume isotropic and homogeneous blood perfusion rates to keep the model simple and computationally efficient. The finite difference method (FDM) was employed to solve Pennes’ model. Furthermore, differential evolution (DE) [24,25,26,27], a stochastic-heuristic algorithm, was used as an optimization method to minimize damage to healthy tissue and maximize damage to the tumor.

Since solving a partial differential equation (PDE) in a three-dimensional domain requires significant computational time, even with simplified models, combining DE with Pennes’ three-dimensional model significantly impacts performance. To reduce computational time and obtain solutions within a reasonable timeframe, we used general-purpose computing on graphics processing units (GPGPU) via the Compute Unified Device Architecture (CUDA) parallel computing platform to parallelize the implementation of the bioheat model [11,28,29,30], i.e., minimize the time spent evaluating the objective function.

We organize this paper as follows. Section 2 describes the bioheat model, its numerical approximation, the new optimization strategy, and its parallel implementation. The results are presented in Section 3 and are discussed in Section 4. Finally, Section 5 concludes the work and presents plans for future work.

## 2. Material and Methods

### 2.1. Mathematical Model

Pennes’ equation is a well-known mathematical model that represents heat propagation in living tissues. The main characteristics of Pennes’ equation are the simplicity of its computational implementation and the low computational cost required for its numerical resolution.

Equation (1) presents the modified Pennes’ equation used in this work to include the hyperthermia treatment as the heat source:

(1) $$\left\{\begin{array}{cc} \rho c\frac{\partial T}{\partial t}=\nabla \cdot k\nabla T+\omega_{b}\rho_{b}c_{b}\left(T_{a}-T\right)+Q_{m}+Q_{r} & \mathrm{in}\;\Omega \times I,\\ k\nabla T\cdot \vec{n}=0 & \mathrm{on}\;\partial \Omega \times I,\\ T(\cdot ,0)=37 & \mathrm{in}\;\Omega , \end{array}\right.$$

where $\Omega \subset R^{3}$ is the spatial domain, $I\subset R^{+}$ is the time domain, $T:\Omega \times I\to R^{+}$ is the tissue temperature field; ρ, *c*, and *k* are density, specific heat and thermal conductivity of the tissue, respectively; $\rho_{b}$, $c_{b}$, and $\omega_{b}$ are density, the specific heat of the blood and blood perfusion, respectively; $T_{a}$ is the blood temperature; and $Q_{m}$ and $Q_{r}$ are the metabolic heat source and the heat generated by the hyperthermia treatment, respectively.

The following simplifications are considered when using Pennes’ model [9]:

- Equilibrium site: The heat transfer between blood and tissue occurs in capillaries;
- Blood perfusion: The blood flow in capillaries is considered isotropic;
- Vascular architecture: The local vascular geometry is not considered;
- Blood temperature: The body core temperature is the same as that reached by the capillaries.

The specific absorption rate (SAR) [31], denoted by $Q_{r}$, is responsible for the heat generated by the hyperthermia injections, as modeled by Equation (2):

(2) $$Q_{r}=\sum_{i=1}^{N_{p}}Ae^{-r{\left(\vec{x}\right)}_{i}^{2}/r_{0,i}^{2}},$$

where $N_{p}$ is the number of injections points in the tissue; *A* is the energy maximum strength of the volumetric heat generation rate, $r{\left(\vec{x}\right)}_{i}^{2}$ is the distance to the injection point, i.e., $r=\left|\right|\vec{x}-\vec{x_{0}}{\left|\right|}_{2}$; $x_{0}$ is the injection position; and $r_{0}$ is the radius of coverage of hyperthermia. The values of parameters *A* and $r_{0}$ are an estimation derived from experimental data, which describes the properties of ferromagnetic fluid when it is subjected to a magnetic field. The amount of fluid injected at the point of injection, such as 0.1 or 0.2 cc, mainly affects these parameters. Therefore, the efficacy of thermal ablation treatment for tumor cells is heavily reliant on the number of injection points and the values of parameters *A* and $r_{0}$ as described above.

### 2.2. Numerical Scheme

The numerical method employed to solve Equation (1) is the Finite Difference Method (FDM) [32]. We consider the closed domain Ω discretized into a set of regular points defined by $S_{s}=\left\{\left(x_{i},y_{j},z_{k}\right);\;i=0,1,\cdots ,N_{x};\;j=0,1,\cdots ,N_{y};\;k=0,1,\cdots ,N_{z}\right\}$, where $N_{x}$, $N_{y}$, and $N_{z}$ are the number of intervals of length $h_{x}=h_{y}=h_{z}=h$. Moreover, the time domain *I* is partitioned into $N_{t}$ equal time intervals of length $h_{t}$, i.e., $S_{t}=\left\{\left(t_{n}\right);n=0,1,\cdots ,N_{t}\right\}$. To obtain the discrete form of the model, we employ a Forward-Time Central-Space (FTCS), resulting in an explicit numerical method. This scheme has convergence order $O(h^{2},h_{t})$.

Equation (3) presents the discretization, considering a heterogeneous medium, of the Pennes’ model described in Equation (1):

(3) $$\begin{array}{cc} T_{i,j,k}^{n+1}=\frac{h_{t}}{\rho c}[ & \frac{k_{i+1/2,j,k}\left(T_{i+1,j,k}^{n}-T_{i,j,k}^{n}\right)-k_{i-1/2,j,k}\left(T_{i,j,k}^{n}-T_{i-1,j,k}^{n}\right)}{h^{2}}+\\ & \frac{k_{i,j+1/2,k}\left(T_{i,j+1,k}^{n}-T_{i,j,k}^{n}\right)-k_{i,j-1/2,k}\left(T_{i,j,k}^{n}-T_{i,j-1,k}^{n}\right)}{h^{2}}+\\ & \frac{k_{i,j,k+1/2}\left(T_{i,j,k+1}^{n}-T_{i,j,k}^{n}\right)-k_{i,j,k-1/2}\left(T_{i,j,k}^{n}-T_{i,j,k-1}^{n}\right)}{h^{2}}+\\ & \rho_{b}c_{b}\omega_{b}\left(T_{a}-T_{i,j,k}^{n}\right)+Q_{m}+Q_{r}]+T_{i,j,k}^{n}, \end{array}$$

where $k_{i+1/2,j,k}$ is the thermal conductivity evaluated at the midpoint. In this paper, we considered a piecewise homogeneous media where the thermal conductivity is a discontinuous function, so the thermal conductivity can be estimated using the harmonic mean, i.e.,

(4) $$k_{i+1/2,j,k}\approx \frac{2k_{i,j,k}k_{i+1,j,k}}{k_{i,j,k}+k_{i+1,j,k}}.$$

Equation (4) assures the continuity of the flux. Furthermore, the thermal conductivity for the other midpoints is evaluated using the same idea.

### 2.3. Differential Evolution

Differential evolution is a stochastic-heuristic algorithm used in optimization problems, i.e., used to find a value that minimizes or maximizes an objective function [33]. DE uses concepts from biological evolution to improve iteratively the results obtained by the use of candidate solutions. These candidate solutions represent a population whose best individuals are selected to pass their characteristics to their offspring. Mutations can occur during this process, changing the characteristics inherited.

The algorithm creates a random population, called the parent population, with a fixed number of individuals. A new generation is then created based on the parent population using three evolutionary operators, mutation, crossover, and selection, replacing the parent generation. A stopping criterion defines when the loop stops. An error below a given threshold or a maximum number of iterations are examples of stopping criteria. Equation (5) illustrates the stopping criterion adopted in this work:

(5) $$\sigma \left(O\left(p\right)\right)\leq atol+tol*\left|\bar{O(p)}\right|,$$

where σ(O(p)) is the standard deviation of the objective function for the population, atol and tol are absolute and relative tolerance for convergence, respectively, and $\left|\bar{O(p)}\right|$ is the absolute mean value of the objective function for the population.

The objective function to be minimized considers the volumes of tumor and healthy tissue affected by the hyperthermia treatment. More specifically, partial and total damage of tumor tissue cause a positive impact on the function, while healthy tissue damage represents a penalty, as shown in Equation (6):

(6) $$\min O\left(p\right)=300-N_{t}-\left(100-N_{h}\right)-100\beta ,$$

where *p* is the set of points to be estimated, $N_{t}\in \left[0,100\right]$ is the percentage of tumor tissue damage, and $N_{h}\in \left[0,100\right]$ is the percentage of healthy tissue damage. β∈{0,1} is a variable that equals 1 when the entire tumor reaches $43{\;}^{{}^{\circ}}$C or more and 0 otherwise.

Since $T\geq 43{\;}^{{}^{\circ}}$C is considered a target temperature for tissue damage, the proposed objective function aims to locate the best position for injecting the nanoparticles to increase the temperature in the tumor tissue up to $43{\;}^{{}^{\circ}}$C or higher while keeping the healthy tissue temperature below $43{\;}^{{}^{\circ}}$C as much as possible. Therefore, $N_{t}$ represents the percentage of tumor tissue with $T\geq 43{\;}^{{}^{\circ}}$C, and $N_{h}$ represents the percentage of healthy tissue with $T<43{\;}^{{}^{\circ}}$C.

A new value is generated using a particular mutation strategy during the mutation operation stage. This work adopts *best/1/bin* as the mutation strategy. This strategy uses the best solution found in the parent population to produce the mutation vector $V_{p}$ as shown in Equation (7):

(7) $$V_{p}^{i}=X_{best}^{i}+F*\left(X_{a}^{i}-X_{b}^{i}\right).$$

The next step uses the crossover operation to introduce more mutations into the population, mixing the mutation vector obtained in the previous step with a target vector $X_{r}$. The idea is to increase the diversity of the trial vector, composed of individuals that will be evaluated in the final step. The crossover operation works as follows. A random number is drawn for each position of the trial vector $U^{i}$. If this number is greater than the crossing constant *C*, that position of the trial vector receives the equivalent position of the target vector. Otherwise, the value is taken from the mutation vector, i.e.,

(8) $$U^{i}=\left\{\begin{array}{cc} V_{p}^{i}, & \;\mathrm{if}\;\;r_{i}\leq C\\ X_{r}^{i}, & \;\mathrm{otherwise}. \end{array}\right.$$

Finally, each value of the trial vector is used to evaluate the objective function. Then, the result is compared to the target vector. The best individual, i.e., the one that generated the error closer to zero, is selected to be part of the next generation.

(9) $$X^{i+1}=\left\{\begin{array}{cc} U_{p}^{i}, & \;\mathrm{if}\;\;O\left(U_{p}^{i}\right)\leq O\left(X_{r}^{i}\right)\\ X_{r}^{i}, & \;\mathrm{otherwise}. \end{array}\right.$$

### 2.4. CUDA Parallel Programming

In this paper, differential evolution used Pennes’ model to evaluate its objective function. Unfortunately, the sequential computation time required to solve one generation of the differential evolution is prohibitive: it takes circa 864 minutes to complete. About 98% of this time is spent solving Pennes’ bioheat model, i.e., about 851 minutes. A parallel strategy based on CUDA’s SIMT (Single Instruction, Multiple Threads) programming model was implemented to reduce this execution time. More specifically, the FDM was implemented as a CUDA kernel, i.e., a piece of code that the CPU, called the host, can invoke to be executed on the GPU, also called the device.

Due to their separate memory spaces, some steps must be followed by programmers that wish to have their codes executed on the device. First, memory must be allocated on the device to store the data it will process. After that, data that the kernel will use must be copied from the host to the device. The host can then call the kernel on the device. Finally, after the kernel finishes its execution, the host must copy the results back from the device to have access to them.

**Figure 1:**
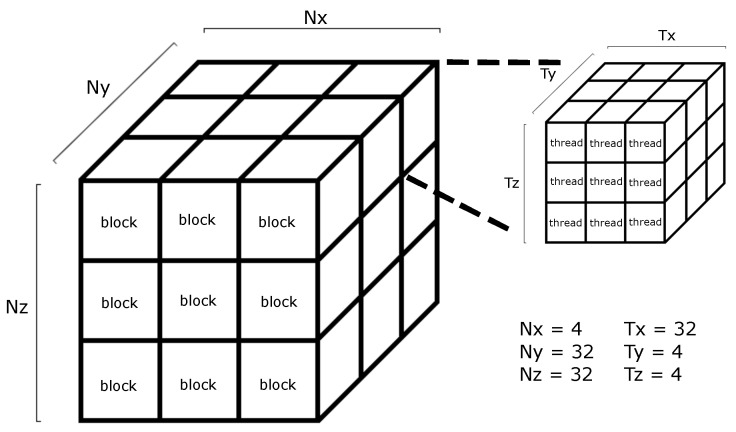
Scheme illustrating the blocks and threads organization used in the simulations of this study. The dimensions were computed by the occupancy calculator (see Figure 2).

When a kernel is launched, it is also necessary to define how threads organize themselves to execute the kernel code. The programmer defines a grid and block sizes for this purpose. The grid size tells the GPU the number of kernel instances it must launch. On the other hand, the block size specifies how many threads must be created to execute each kernel instance. Threads in blocks and blocks in grids are organized in a one-dimensional, two-dimensional, or three-dimensional way. Our implementation creates a three-dimensional grid, and each element of this grid has one three-dimensional block, as depicted in Figure 1. Each thread computes data related to its position in the domain.

**Figure 2:**
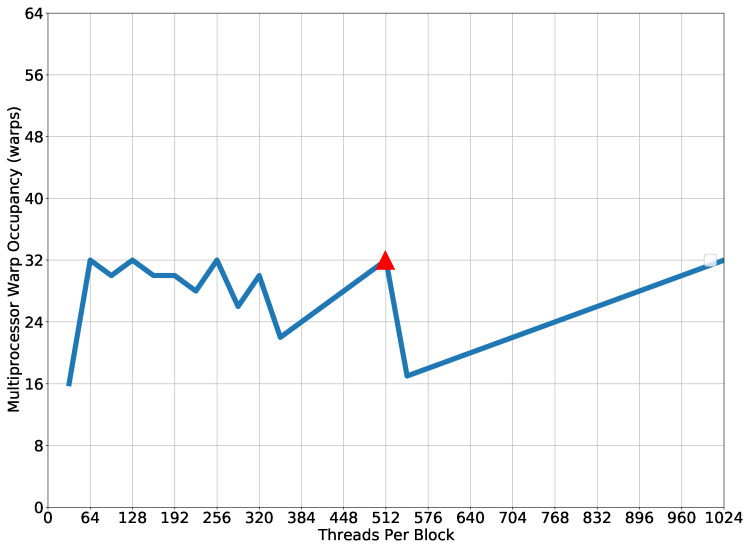
Results obtained by the use of the CUDA Occupancy Calculator. The Figure shows the consequence of varying the number of threads per block in the GPU occupancy. The idea is to choose the block size that maximizes GPU occupancy. The same occupancy can be achieved by distinct configurations. The red triangle represents the block size used in this work.

The execution of a code on the GPU requires adaptations and optimizations to improve performance since the internal GPU architecture is very distinct from the CPU architecture. The usual CUDA optimizations implemented by those that execute code on the GPU are also present in our code, such as the replacement of statements by ternary operators, the substitution of traditional functions by macros and inline functions, and the use of thread’s register space to store data accessed multiple times to reduce memory bandwidth usage. Additionally, the optimal values for the thread block size were computed using the CUDA Occupancy calculator, as shown in Figure 2. These values were calculated to maximize the occupancy of the stream multiprocessors. Maximizing the occupancy can help to mask the latency during some memory operations.

## 3. Numerical Results

### 3.1. Computational Environment

The numerical model and optimization method presented in Section 2 were implemented by the authors using the C programming language. The sequential version of the code was compiled using gcc version 11.3.1 with the optimization flag −O3 enabled. The CUDA version of the code was compiled using nvcc version 11.7.64, with the optimization flag −O3 enabled as well. Both the sequential and parallel versions of the code were executed on a 2.90 GHz Intel^®^  Core^TM^i7-10700 CPU processor running Linux version 5.17.4-200.fc35.x86_64 and equipped with a GeForce GTX 1650 Super Turing architecture. The CPU has 16 hyper-threading cores, but only eight physical cores. The GPU has 1280 CUDA cores and 4 GB of GDDR6 memory. Both the sequential and parallel versions of the code ran on a single CPU core. Finally, the simulations were post-processed using ParaView version 5.10.1.

### 3.2. Simulation Scenarios

This work evaluated the optimization method developed in this work using three distinct simulation scenarios. The optimization method aims to find the sites that maximize tumor damage while minimizing the healthy tissue affected during the hyperthermia treatment. The results obtained by the proposed optimization strategy were compared to those of a naive approach that always injects the nanoparticles in the middle of the tumor. In all simulation scenarios, a three-dimensional cubic domain with lengths equal to 0.1 m represents the tissue, and spherical with radii of 0.01 m represents the tumors. As the next section shows, a grid independence study determined the mesh size used in this work: $N_{x}=N_{y}=N_{z}=128$. The time step used was $h_{t}=0.1$. We simulated a single hyperthermia treatment session of 50 min.

The first scenario considers a single tumor located in the center of the domain and a single injection point. The second scenario considers two tumors in the domain and two injection points. The third scenario considers three tumors and two or three injection points.

Almost all parameters are common to all scenarios. Table 1 presents the parameter values that represent healthy and tumorous tissues. The missing parameter, $N_{p}$, changes its value according to the considered scenario.

The optimization strategy was executed using different seeds to certify that the algorithm did not converge to a local minimum. The DE population size is proportional to the number of parameters to be adjusted ($N_{param}$), i.e., $N_{param}\times pop$ where we considered pop=35 for all scenarios. Moreover, if the simulation does not reach the convergence criterion after 10,000 steps, the DE stops considering this optimization attempt unsuccessfully.

### 3.3. Grid Independence Study

The first scenario was used to execute a grid independence study. A grid independence study is a systematic investigation to determine the numerical solution’s sensitivity to the size of the computational grid. It involves evaluating the solution of a problem using different grid sizes to determine the point at which the solution becomes insensitive to the grid size. The grid independence study is an essential step in numerical simulations to ensure the accuracy and reliability of the results.

The grid independence study was performed using the first scenario, starting with a mesh size of $N_{x}=N_{y}=N_{z}=256$. We observed that the healthy tissue affected was equal to 1.49% for the best individual. Then, we reduced the mesh size to $N_{x}=N_{y}=N_{z}=128$. For this mesh size, we observed that the healthy tissue damage was equal to 1.53% for the best individual, i.e., a small variation of 0.04%. We adopted the mesh size of $N_{x}=N_{y}=N_{z}=128$ for all three scenarios because this coarse-grained mesh allows us to obtain accurate and reliable numerical solutions while minimizing computational costs.

### 3.4. Results of the Optimization Method

Table 2, Table 3 and Table 4 present, for each scenario, the best individuals found for ten executions of the optimization method. These tables present the position suggested for the nanoparticle injection and the value of its objective function O(p) (Equation (6)). In this optimization problem, the best individual is the one that obtains an O(p) value closer to 0. The last two lines show the mean and standard deviation values for each column, respectively.

#### 3.4.1. First Scenario

**Figure 3:**
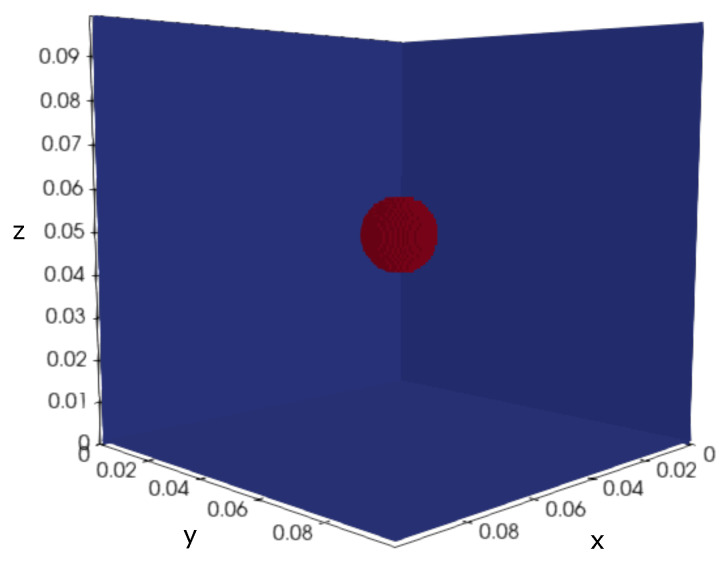
This figure illustrates the tissue used in the first scenario. The red sphere represents the tumor, and the blue area represents the healthy tissue.

The first scenario considers a tumor centered at (0.050,0.050,0.050), i.e., in the center of the domain (as shown in Figure 3). The DE tries to find a single injection point for the nanoparticles that can kill all tumor cells, and at the same time minimize the number of healthy cells killed. This scenario considers the values shown in Table 1 and $N_{p}=1$ to solve the Equation (1). The points found, for each execution, are presented in Table 2.

**Figure 4:**
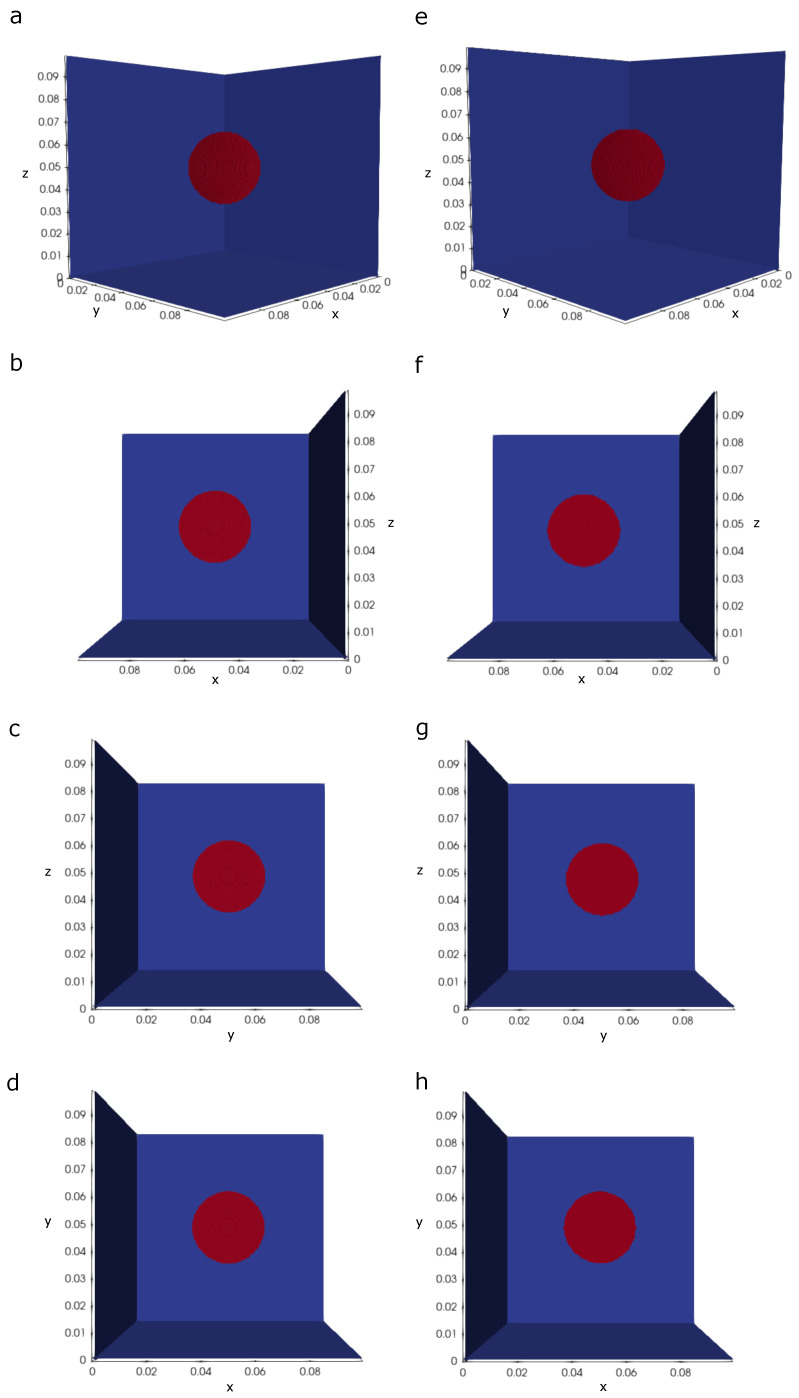
Results for the first scenario are presented in (**a**–**h**), showing different views of tissue damage using the results of Equation (1) at t=50 min. The red area denotes tissue that reached $T\geq 43{\;}^{{}^{\circ}}$C, i.e., the accepted temperature for thermal damage in various tissues. The left panel (**a**–**d**) considers P1=(0.050,0.050,0.050), i.e., the naive solution. The right panel (**e**–**h**) considers P1=(0.050408,0.050745,0.048888), i.e., the injection point suggested by the optimization method. As can be observed, no visual difference is noticeable between the two strategies.

Figure 4 presents different views of the simulation results. They represent the damages due to one 50 min section of the hyperthermia treatment. The panels on the left column of Figure 4a–d are the results of the naive approach of injecting the nanoparticles in the middle of the tumor, while the right column of the Figure presents the results obtained when the best result of Table 2 is considered, i.e., when the nanoparticles are injected at the coordinates given by line 5 (0.050408,0.050745,0.048888). In this scenario, the results of the naive and optimized approach were very similar: in both cases, the total tissue damage of the tumor was observed, and 1.53% of healthy tissue was damaged.

**Figure 5:**
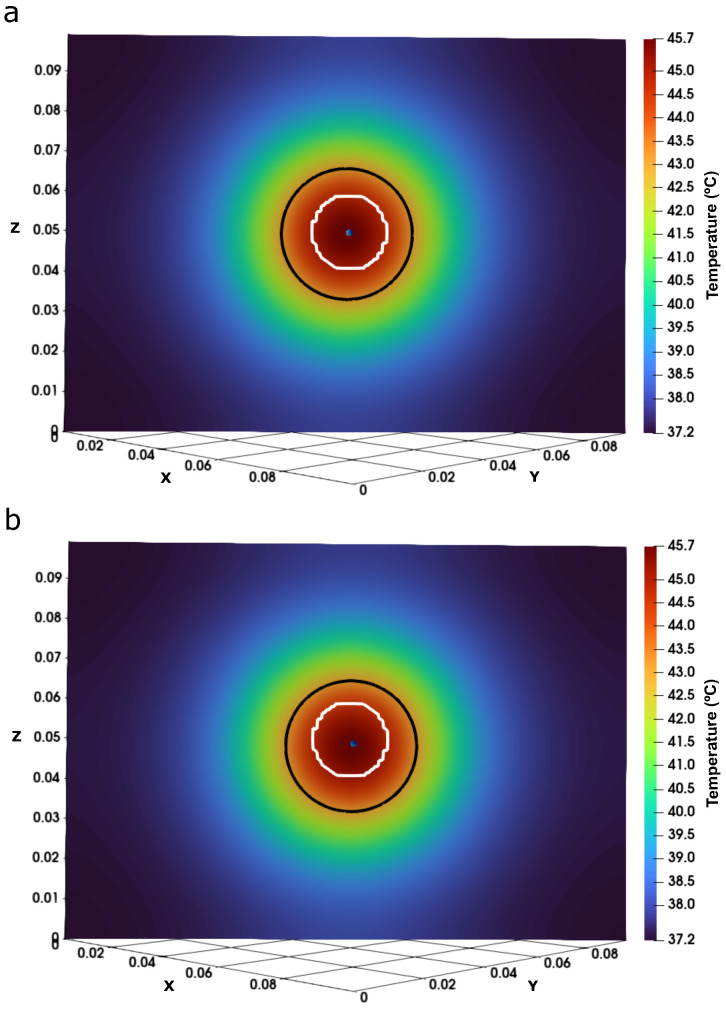
Heatmap of the model solution (Equation (1)) in the plane x=−y at t=50 min. The solid black contour highlights the portion of the domain that reaches $T\geq 43{\;}^{{}^{\circ}}$C. The solid white contour represents the tumor location, and the blue dot is the position of the nanoparticle injection. (**a**) presents the injection positioned in the center of the tumor (P1=(0.050,0.050,0.050)), while (**b**) represents the injection in the point suggested by the optimization method (P1=(0.050408,0.050745,0.048888)).

The damage caused by the treatment can be better observed in Figure 5, which presents the heatmap of the model solution in the plane x=−y at t=50 min. The naive (**a**) and the optimized (**b**) solutions are presented in Figure 5.

#### 3.4.2. Second Scenario

**Figure 6:**
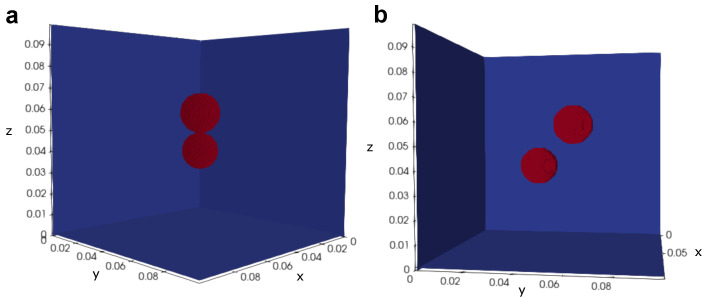
This figure illustrates the tissue used in the second scenario. The red spheres represent the tumors, and the blue area represents the healthy tissue. (**a**) shows the x = y view and (**b**) the x = 0 view.

The second experiment considered two injection points and two tumors. One tumor was centered at (0.040,0.040,0.040), and the other was centered at (0.060,0.060,0.060), as shown in Figure 6. This second scenario considers the healthy and tumorous tissue properties, this time using the values presented in Table 1 and $N_{p}=2$ as parameters to the algorithm described in Section 2. The injection points found by the algorithm are shown in Table 3.

**Figure 7:**
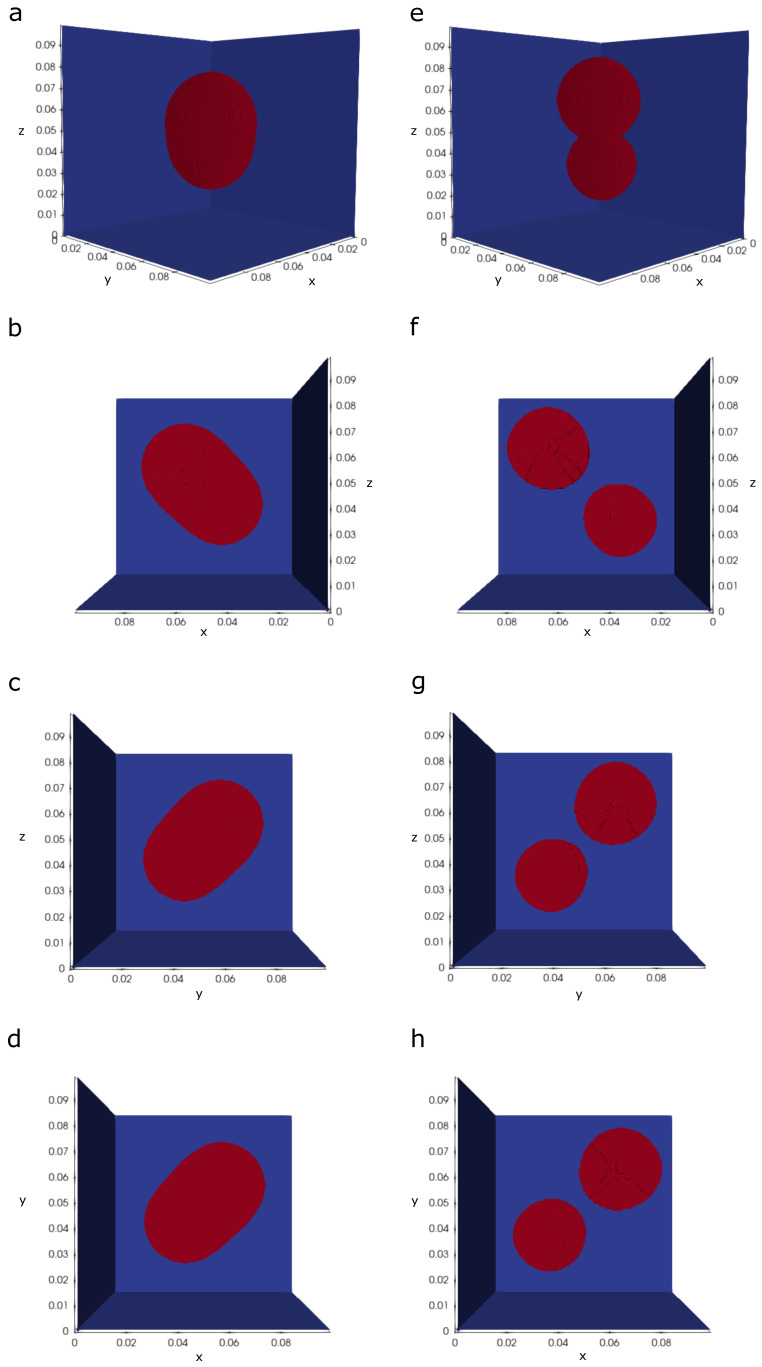
Results for the second scenario. (**a**–**h**) present different views of tissue damage using the results of Equation (1) at t=50 min. The red area denotes the tissue that reached $T\geq 43{\;}^{{}^{\circ}}$C. The left panel (**a**–**d**) considers $P_{1}=\left(0.040,0.040,0.040\right)$ and $P_{2}=\left(0.060,0.060,0.060\right)$, i.e., the naive solution. The right panel (**e**–**h**) considers $P_{1}=\left(0.033357,0.034644,0.033353\right)$ and $P_{2}=\left(0.066553,0.065626,0.066493\right)$, i.e., the injection point suggested by the optimization method.

Figure 7 presents different views of the simulation results. They represent the damages due to one 50 min section of the hyperthermia treatment. The panels on the left column of Figure 7a–d are the results of the naive approach of injecting the nanoparticles in the middle of the two tumors, while the right column of the Figure presents the results obtained when the best results of Table 3 are considered, i.e., when the nanoparticles are injected at the coordinates given by line 10 ((0.033357, 0.034644, 0.033353) and (0.066553, 0.065626, 0.066493)). This time, the results of the naive and optimized approach were distinct. The naive version resulted in 7.03% of healthy tissue damage, while the optimized version resulted in 4.71% of damage, a significant reduction of 33%. Both the optimized and naive versions resulted in total damage to the tumor tissue.

**Figure 8:**
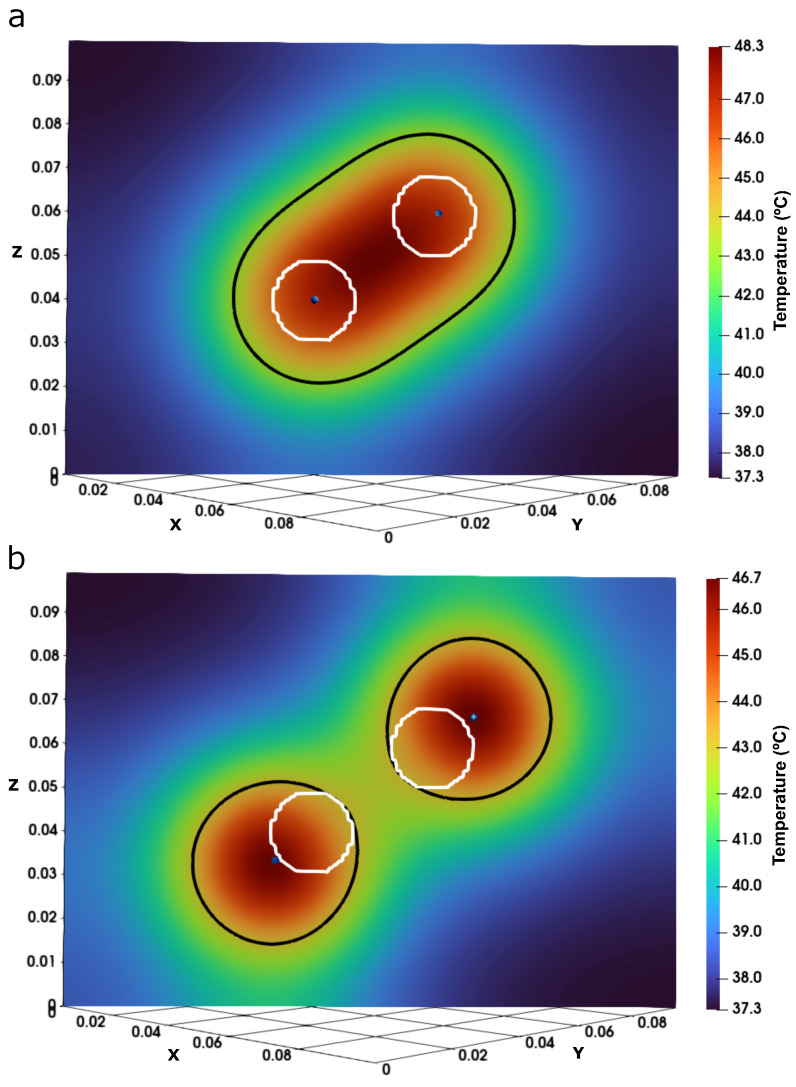
Heatmap of the model solution (Equation (1)) in the plane x=−y at t=50 min. The solid black contour highlights the portion of the domain that reaches $T\geq 43{\;}^{{}^{\circ}}$C. The solid white contour represents the tumor locations, and the blue dot is the position of the nanoparticle injections. (**a**) presents the injection positioned in the center of the tumors ($P_{1}=\left(0.040,0.040,0.040\right)$ and $P_{2}=\left(0.060,0.060,0.060\right)$), while (**b**) represents the injection in the point suggested by the optimization method (P1=(0.033357,0.034644,0.033353) and P2=(0.066553,0.065626,0.066493)).

Figure 8 presents the damage caused by the treatment as a heatmap of the model solution in the plane x=−y at t=50 min. The naive (**a**) and the optimized (**b**) solutions are shown in Figure 8, which delimits the portion of the domain that reaches $T\geq 43{\;}^{{}^{\circ}}$C, and presents the tumor sites and injection points. In both cases, one can observe that the isoline $T=43{\;}^{{}^{\circ}}$C includes the whole tumor.

#### 3.4.3. Third Scenario

**Figure 9:**
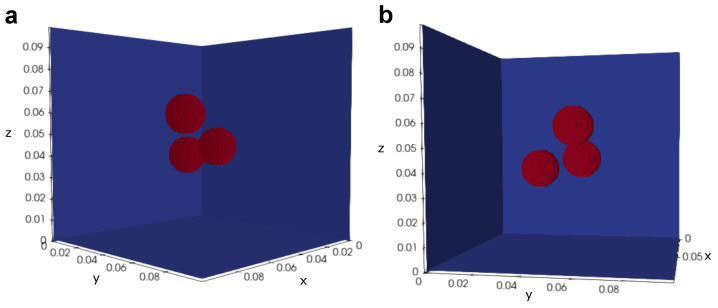
This figure illustrates the tissue used in the third scenario. The red sphere represents the tumor, and the blue area represents the healthy tissue. Both figures show different views of the same simulated domain. Figure (**a**) shows the x=y view and Figure (**b**) the x=0 view.

The last experiment considers three tumors, which are centered at (0.045,0.035,0.040), (0.045,0.055,0.045), and (0.065,0.055,0.060), as depicted in Figure 9. The first attempt for the optimization in the third scenario was using $N_{p}=3$, i.e., searching for the three best injection points localizations. However, the optimization algorithm pushes one of the three points away from the tumor, in the domain’s borders, or, in other words, out of the simulated tissue. Thus, we tried to use $N_{p}=2$, and the algorithm obtained less damage to the healthy tissue, and at the same time, the tumor site reached a temperature of 43${\;}^{{}^{\circ}}$C or higher. So, although three tumors are present, the optimization considers only two injection points, while the naive approach keeps considering one injection for each tumor, i.e., three injection points. The third scenario uses the values shown in Table 1 and $N_{p}=2$ or $N_{p}=3$ to solve Equation (1). The points found by the DE are presented in Table 4.

**Figure 10:**
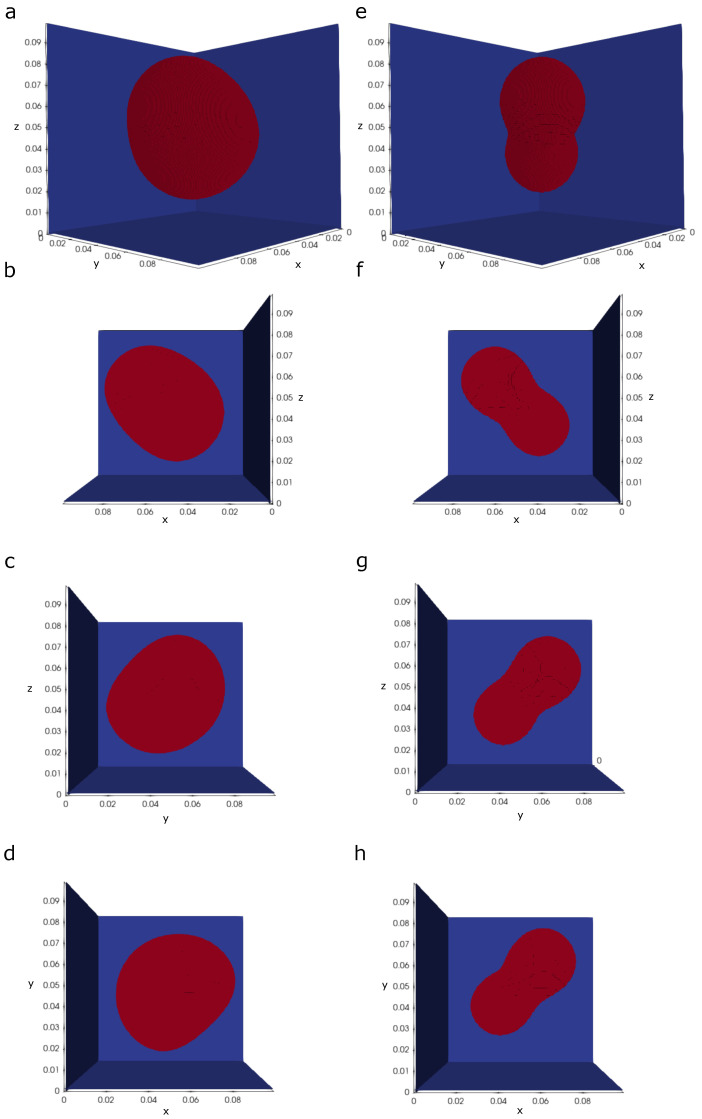
Results for the third scenario. (**a**–**h**) present different views of tissue damage using the results of Equation (1) at t=50 min. The red area denotes the tissue that reached $T\geq 43{\;}^{{}^{\circ}}$C. The left panel (**a**–**d**) considers $P_{1}=\left(0.045,0.035,0.040\right)$, $P_{2}=\left(0.045,0.055,0.045\right)$ and $P_{3}=\left(0.065,0.055,0.060\right)$, i.e., the naive solution. The right panel (**e**–**h**) considers $P_{1}=\left(0.035855,0.039716,0.038324\right)$ and $P_{2}=\left(0.061546,0.065134,0.063371\right)$, i.e., the injection point suggested by the optimization method.

**Figure 11:**
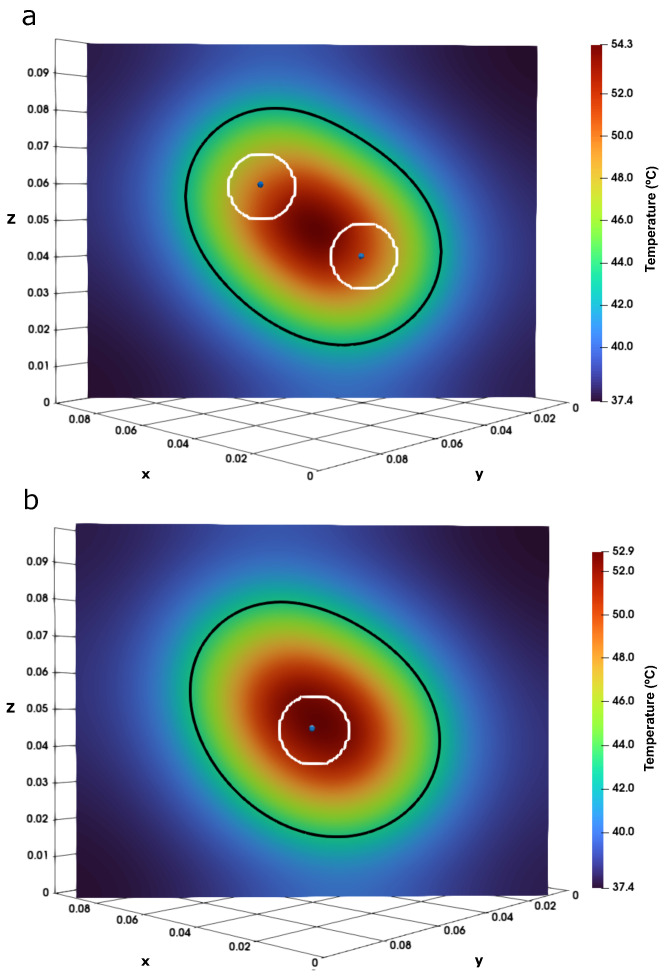
Heatmap of the model solution (Equation (1)) in the plane x=−y at t=50 min for the naive approach. The solid black contour highlights the portion of the domain that reaches $T\geq 43{\;}^{{}^{\circ}}$C. The solid white contour represents the tumor locations, and the blue dot is the position of the nanoparticle injections. (**a**) presents the injection positioned in the center of the tumors ($P_{1}=\left(0.045,0.035,0.040\right)$ and $P_{2}=\left(0.065,0.055,0.060\right)$), while (**b**) presents the injection in the center of the tumor $P_{3}=\left(0.045,0.055,0.045\right)$.

**Figure 12:**
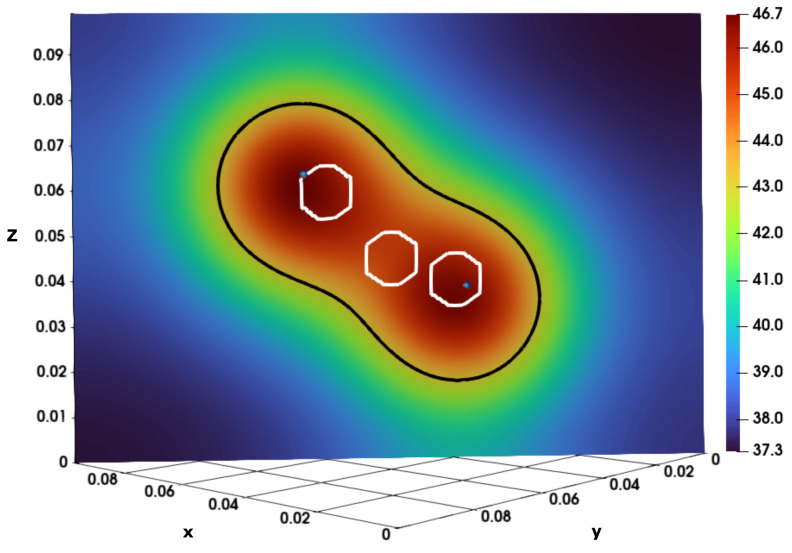
Heatmap of the model solution (Equation (1)) in the plane x=−y at t=50 min for the point suggested by the optimization method (P1=(0.035855,0.039716,0.038324) and P2=(0.061546,0.065134,0.063371)). The solid black contour highlights the portion of the domain that reaches $T\geq 43{\;}^{{}^{\circ}}$C. The solid white contour represents the tumor locations, and the blue dot is the position of the nanoparticle injections.

Figure 10 presents different views of the simulation results. They represent the damages due to one 50 min section of the hyperthermia treatment. The panels on the left column of Figure 10a–d are the results of the naive approach of injecting the nanoparticles in the middle of the three tumors, while the right column of the Figure presents the results obtained when the best results of Table 4 are considered, i.e., when the nanoparticles are injected at the coordinates given by line 10 ((0.035855, 0.039716, 0.038324) and (0.061546, 0.065134, 0.063371)). Again, the results of the naive and optimized approaches were distinct. The naive version resulted in 14.05% of healthy tissue damage, while the optimized version resulted in 5.78% of damage, a significant reduction of about 59%. As occurred in the first and second scenarios, both the optimized and naive versions resulted in total damage to the tumor tissue, as can be observed in the heatmaps of Figure 11 and Figure 12. This time we opt to show the results of the naive and optimized approaches in separate figures because the naive version cannot show all injection points in a single heatmap.

### 3.5. Performance Evaluation

This section evaluates the performance of the parallel version of the code, using for this purpose the first scenario executed with three different mesh sizes: (a) N=64; (b) N=128; and (c) N=256, where $N=N_{x}=N_{y}=N_{z}$.

**Figure 13:**
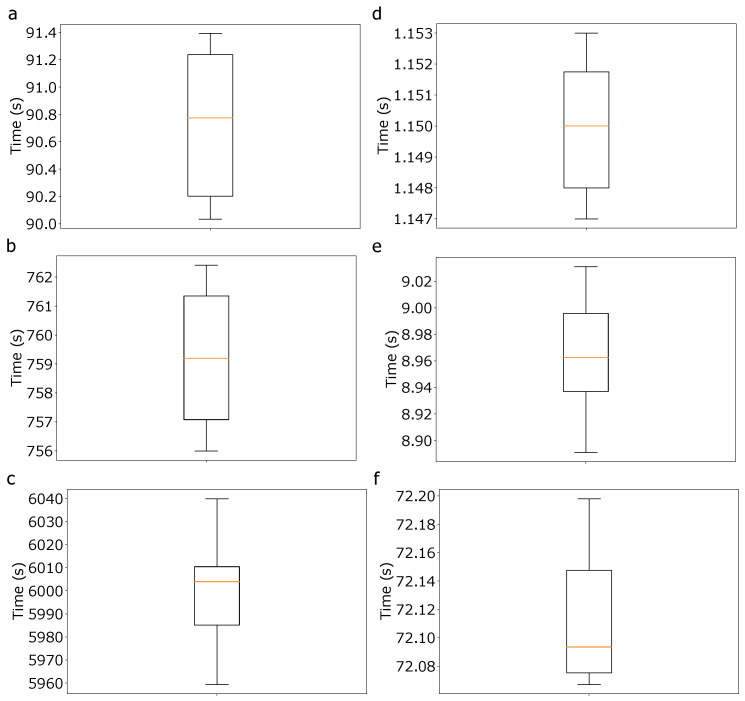
Boxplot for ten executions for distinct discretizations. The first, second, and third lines represent the execution time for N=64, N=128, and N=256, respectively. The first column (**a**–**c**), presents the execution time for the sequential version of the code, while the second column (**d**–**f**), presents the execution time for parallel one.

As shown in Section 2, the total execution time of the code is dominated by the resolution of Pennes’ model. For this reason, the speedups reported in Table 5 refer only to the execution of a single call of this function. The execution times reported in this Table are the mean of 10 executions, with a confidence interval of 95%. Figure 13 presents the boxplot for these ten sequential and parallel executions, considering the three mesh sizes used in the performance evaluation.

As one can observe from the figures presented in Table 5, the parallel implementation was very effective to improve performance: a minimum speedup of 82 times was achieved.

## 4. Discussion

In all simulated scenarios, for both the naive and optimized strategies, the tumor damage reached 100%. The main difference between the strategies was the reduction in healthy tissue damage: the proposed algorithm reduced damage by up to 33% in the second scenario and 59% in the third. In the case of the first scenario, similar results were obtained, as can be observed in Figure 4: no visual difference is noticeable between the naive strategy and the optimized one. These numerical experiments suggest that the proposed method is better suited for controlling healthy tissue damage in hyperthermia treatment since it suggests non-intuitive sites for injecting the nanoparticles.

The results shown in Table 2, Table 3 and Table 4 show that this strategy is robust for different numbers of tumors once all executions of the algorithm converge to a viable solution. Moreover, all absolute standard deviations (SD) of the injection points positions are lower than $10^{-2}$, and the SD of healthy tissue damage is only 0.0189.

The main disadvantage of the proposed method is the time required for its execution. The function that implements the DE optimization has to call the function that computes Pennes’s algorithm multiple times, and this function demands large amounts of time to finish its work. The proposed solution to solve this issue is to implement a parallel version of the code using CUDA, which was very effective in reducing the execution time: speedups up to 84 times were observed. This speedup allows us to use more refined meshes, whose parallel execution time is lower than the sequential execution time of the coarse mesh. The use of refined meshes contributes to improving the quality of simulations.

This paper presents some limitations. The model used to represent heat propagation adopts some simplifications on purpose: to reduce the cost of evaluating the objective function of the differential evolution method thousands of times. These simplifications could give way to other methods. For example, this paper considered a target temperature of $T\geq 43{\;}^{{}^{\circ}}$C as the threshold for tissue damage and induction of cell necrosis which is considered a threshold temperature to induce cell necrosis within a reasonable duration. In our experiments, we simulated 50 min of hyperthermia treatment, but the duration was not considered a parameter of hyperthermia success. Additionally, at this temperature, there is a delay in achieving tissue damage, which was not considered in this paper. Moreover, there are more precise ways to quantify tumor ablation, such as the Arrhenius models [36,37], or considering a temperature-dependent time delay, such as the Pearce model [38,39]. Furthermore, the blood perfusion rate ($\omega_{b}$) was considered constant, but recent studies have analyzed its dependencies on blood temperature [11] and thermal damage [36]. It is also possible to include the thermal bystander effect [40] in the model. In addition, there are different ways to model the external heat source due to the nanoparticle injections ($Q_{r}$ in Equation (1)), such as the one described in Singh [41]. Still, we opt for the model used by Salloum et al. [31] due to its simplicity. These changes would increase the time required to calculate the objective for each individual in differential evolution, impairing performance. On the other hand, the use of simplifications allowed us to obtain the optimization results in 72 s when using the parallel version of the code. Finally, it is necessary to note that this study is based on a theoretical model and may not fully represent the complex biological processes observed in real tumors. Additionally, the results of this study may not be directly applicable to clinical settings, and further experimental validation is required.

Numerous studies propose administering multiple injections into tumors to facilitate even heating throughout the tumor area [42]. Multi-injections could be considered a feasible choice for tumors located near the skin, where the likelihood of further tumor spread (metastasis) is minimal to negligible. On the other hand, some studies suggest that multi-injections in deep tissue might lead to tumor spread [41]. Therefore, there is no consensus on the viability of using multi-injections.

## 5. Conclusions and Future Works

This work proposes a strategy for planning the injection sites of magnetic nanoparticles in hyperthermia treatments. The proposed method uses an optimization algorithm, differential evolution, to define injection points that maximize tumor tissue damage while minimizing damage to healthy tissue. Simulation results show that this strategy reduced healthy tissue damage by up to 59% compared to the naive scenario of injecting nanoparticles in the middle of the tumor, in contrast to the non-trivial positions suggested by the algorithm in the third scenario (see Section 3.4.3). The resolution of the mathematical model in a three-dimensional domain requires a significant amount of time to obtain the best injection points. We chose to keep the model as simple as possible to reduce the computational time required to solve the optimization strategy in an n-dimensional search space associated with the resolution of the set of PDEs in a three-dimensional domain. Additionally, the implementation of a parallel version of the code using CUDA improved performance up to 84 times (see Table 5).

In future work, we plan to evaluate the proposed method for more realistic tumors and tissue shapes [43,44], or even those obtained from patient-specific images [45]. The simplifications adopted in this model may not be the best choices to represent some phenomena. For this reason, we also plan to implement a new version of the code considering anisotropic magnetic nanoparticle distribution, nanoparticle migration, and thermal damage index, comparing its results with our simplified approach [40]. Moreover, when considering several tumors close to each other, the simulation results raised an unexpected question: what is the minimum number of injection points required to remove all tumors? Additionally, we plan to investigate the use of multiple GPUs to solve the PDEs, as this architecture is more commonly available in desktop computers that can be used for treatment planning [46,47,48].

## Figures and Tables

**Table 1:** Parameter values used for all scenarios, for healthy and tumor tissues, to solve Equation (1). The parameters are adapted from the literature [12,34,35].

Parameters	Unit	Healthy Tissue	Tumor Tissue
*k*	W/m ${}^{{}^{\circ}}$C	0.51	0.64
$\omega_{b}$	s${}^{-1}$	$5.0\times 10^{-4}$	$1.25\times 10^{-3}$
ρ	Kg/m${}^{3}$	1000.0	1000.0
$\rho_{b}$	Kg/m${}^{3}$	1000.0	1000.0
$Q_{m}$	W/m${}^{3}$	420.0	4200.0
*c*	J/Kg ${}^{{}^{\circ}}$C	4200.0	4200.0
$c_{b}$	J/Kg ${}^{{}^{\circ}}$C	4200.0	4200.0
*A*	W/m${}^{3}$	$0.08\times 10^{6}$	$0.08\times 10^{6}$
$r_{0}$	m	$1.9\times 10^{-2}$	$1.9\times 10^{-2}$

**Table 2:** Results for 10 executions of the optimization method considering the first scenario. The first three columns show the suggested injection points in X, Y, and Z. The last column shows the value of its objective function. The last two lines show the mean and the standard deviation value for each column.

Optimization	$P_{1}$			O(p)
	$X_{1}$	$Y_{1}$	$Z_{1}$	
1	0.050329	0.048464	0.051144	1.528931
2	0.047534	0.049658	0.052657	1.530411
3	0.049302	0.050300	0.048829	1.529602
4	0.048908	0.051205	0.049924	1.528976
5	0.050408	0.050745	0.048888	1.528015
6	0.050429	0.051157	0.050696	1.528015
7	0.049702	0.049928	0.049644	1.528488
8	0.051778	0.050468	0.048926	1.528595
9	0.050470	0.048895	0.050958	1.529312
10	0.049211	0.051133	0.050279	1.529404
Mean	0.049807	0.050195	0.050195	1.528975
SD	0.001152	0.000958	0.001219	0.000744

**Table 3:** Results for 10 executions of the optimization method considering the second scenario. The first six columns show the two injection points in X, Y, and Z found using the DE algorithm. The last column shows the value of its objective function. The last two lines show the mean and the standard deviation value for each column.

Optimization	$P_{1}$			$P_{2}$			O(p)
	$X_{1}$	$Y_{1}$	$Z_{1}$	$X_{2}$	$Y_{2}$	$Z_{2}$	
1	0.033568	0.033437	0.034166	0.066204	0.066198	0.065880	4.719711
2	0.033158	0.033216	0.034887	0.066381	0.066216	0.064923	4.758118
3	0.034075	0.033586	0.033561	0.065539	0.067372	0.065266	4.730225
4	0.033551	0.034300	0.033462	0.066756	0.065333	0.066400	4.717499
5	0.033578	0.034553	0.033297	0.065525	0.065819	0.067165	4.723206
6	0.034213	0.033093	0.034340	0.066077	0.066589	0.066171	4.719650
7	0.034027	0.033663	0.033887	0.066522	0.066382	0.065932	4.714081
8	0.033460	0.033370	0.034243	0.067113	0.065617	0.065466	4.719421
9	0.033804	0.035086	0.032993	0.066942	0.065411	0.066230	4.736603
10	0.033357	0.034644	0.033353	0.066553	0.065626	0.066493	4.710052
Mean	0.033679	0.033895	0.033819	0.066361	0.066056	0.065993	4.724857
SD	0.000340	0.000692	0.000585	0.000538	0.000627	0.000654	0.013938

**Table 4:** Results for 10 executions of the optimization method considering the third scenario, i.e., three tumor positioned at (0.045,0.035,0.040), (0.045,0.055,0.045) and (0.065,0.055,0.060). The first six columns show the two injection points in X, Y, and Z found using the DE algorithm. The last column shows the value of its objective function. The last two lines show the mean and the standard deviation value for each column.

Optimization	$P_{1}$			$P_{2}$			O(p)
	$X_{1}$	$Y_{1}$	$Z_{1}$	$X_{2}$	$Y_{2}$	$Z_{2}$	
1	0.035316	0.038939	0.037442	0.060828	0.064375	0.062292	5.794724
2	0.036168	0.040316	0.039479	0.061440	0.065741	0.064563	5.820786
3	0.036026	0.039912	0.038796	0.063148	0.064854	0.062461	5.791748
4	0.036452	0.038837	0.036926	0.058517	0.064564	0.064386	5.817810
5	0.035807	0.040034	0.039717	0.062029	0.065424	0.063476	5.831345
6	0.036532	0.040267	0.038953	0.062181	0.065351	0.064058	5.804321
7	0.035845	0.038814	0.036999	0.059579	0.064102	0.063266	5.825226
8	0.036897	0.038718	0.037442	0.059815	0.064756	0.063467	5.833176
9	0.036306	0.039930	0.038255	0.062287	0.064951	0.063020	5.798340
10	0.035855	0.039716	0.038324	0.061546	0.065134	0.063371	5.777054
Mean	0.036120	0.039548	0.038233	0.061137	0.064925	0.063436	5.809453
SD	0.000450	0.000646	0.001005	0.001439	0.000504	0.000747	0.018912

**Table 5:** Execution time of the sequential and parallel versions of the code for distinct meshes sizes, and their speedups, considering the first scenario executed with the naive approach. Speedup is defined as the ratio between sequential and parallel execution times. The reported execution times are the mean for 10 executions, with a confidence interval of 95%.

Mesh ($N_{x}\times N_{y}\times N_{z}$)	CPU Time (s)	GPU Time (s)	Speedup
64×64×64	90.7 ± 0.55	1.1 ± 0.002	82.5
128×128×128	759.2 ± 2.38	9.0 ± 0.04	84.4
256×256×256	5998.96 ± 22.60	72.1 ± 0.05	83.2
